# Enhanced Thermostability of a Fungal Alkaline Protease by Different Additives

**DOI:** 10.1155/2014/109303

**Published:** 2014-07-03

**Authors:** Nilesh P. Nirmal, R. Seeta Laxman

**Affiliations:** ^1^Department of Biochemistry, Faculty of Pharmaceutical Sciences, Burapha University, 169 Bangsaen Road, Saensook, Muang, Chonburi 20131, Thailand; ^2^Division of Biochemical Sciences, National Chemical Laboratory, Pune 411 008, India

## Abstract

A fungal strain (*Conidiobolus brefeldianus* MTCC 5184) isolated from plant detritus secreted a high activity alkaline protease. Thermostability studies of the fungal alkaline protease (FAP) revealed that the protease is stable up to 50°C with 40% residual activity after one hour. Effect of various additives such as sugars, sugar alcohols, polyols, and salts, on the thermostability of FAP was evaluated. Among the additives tested, glycerol, mannitol, xylitol, sorbitol, and trehalose were found to be very effective in increasing the stability of FAP, which was found to be concentration dependent. Fivefold increase in residual activity of FAP was observed in the presence of trehalose (50%) and sorbitol (50%) at 50°C for 4 h, compared to FAP without additive. Other additives like calcium at 20 mM and 10–15% ammonium sulphate showed lower stability improvement than trehalose and sorbitol. NaCl, MgCl_2_, K_2_HPO_4_, and glycine were found to be poor stabilizers and showed only a marginal improvement. PEG 6000 did not show any increase in stability but was found to be slightly inhibitory.

## 1. Introduction

Proteases are one of the largest groups of hydrolytic enzymes having 60% share in world enzyme market. Among them, alkaline proteases have been maximally exploited in food, leather, silk, detergent industries, and waste management. They are also used as important tools in studying the structure of certain oligopeptides, proteins, and polypeptides [1]. However, their overall potential in industrial processes is yet to be fully exploited. The use of enzymes for industrial purpose usually depends on their stability during isolation, purification, storage, and tough operational conditions. Higher thermostability is one of the crucial requirements of an enzyme for its application in industrial processes as it increases the efficiency of enzyme. Therefore, search for thermostable enzymes or enhancing thermostability of enzymes has been the priority of the industries or researchers.

Stabilization of enzymes in soluble form is very important as it is impossible to use insoluble enzymes in several biotechnological applications including detergent, food, cosmetic, and textile industries. Several approaches were carried out to improve stabilization of enzymes in soluble form, including changing the environment by means of additives such as sugars and osmolytes [2], alteration of primary structure of the enzyme by chemical modification [3], protein engineering [4], and introduction of disulphide bridge and covalent immobilization [5], through the formation of a reversible enzyme-inhibitor complex [6]. Addition of stabilizing agents is the simplest and cheapest method to achieve enhanced thermostability of enzymes. Various types of additives have been reported in the literature such as polyols, sugars, metals, surfactants, and salts. New additives are screened every day, which reflect the effectiveness of this method. However, some of these additives may interfere with the final use of the enzyme due to incompatibility with reaction system, mostly in pharmaceutical sector. Despite this, soluble additives were widely practiced in textile, leather, detergent industries, and waste management as a reliable stabilization method. For this study, only those compounds with a well-documented stabilizer for protease were considered. Stabilization by using such additives follows two paths: one by additive-solvent (water) interactions and another by additive-protein interaction. In most of the cases, additive-water interaction favors the thermostability of protein following preferential hydration principle where enzymes get stabilized. On the other hand, in additive-protein interaction most of the proteins are destabilized.

A large proportion of commercially available proteases are derived from* Bacillus* strains [7]. Fungal sources are being increasingly used [8, 9]. Although bacterial proteases have been used in industrial processes long before, the high cost to obtain microbe-free enzyme limited its further promotion. Proteases from fungal origin offer an advantage where the mycelium can be easily removed by filtration. The optimization of fermentation parameters as well as scale up of production of this fungal alkaline protease has been reported earlier [8]. This protease has the potential for application in leather, silk degumming, and detergent industries. The present paper describes the temperature stability of a fungal alkaline protease and its improvement by different additives.

## 2. Materials and Methods

### 2.1. Chemicals

Malt extract, yeast extract, and peptone were obtained from Hi-Media Chemicals, India. Hammerstein casein was obtained from M/s Sisco Research Laboratories, India. Xylitol and trehalose were obtained from Sigma Chemical Co., USA. All other chemicals were of analytical grade.

### 2.2. Microorganism and Enzyme Production

The fungal strain (*Conidiobolus brefeldianus* MTCC 5184) used in the present study was isolated from plant detritus and maintained on MGYP (0.3% malt extract; 1.0% glucose; 0.3% yeast extract; 0.5% peptone) agar slants. Protease was produced in 400 L fermentor in a simple medium containing glucose (2.0%), yeast extract (0.3%), and soybean meal (3.0%) as described by Khandelwal [8]. Inoculum and seed for protease production were developed in 1 L conical flask and 35 L fermentor, respectively. Inoculum was developed by inoculating spores from 2- to-3-day-old MGYP slants in 200 mL GYEP medium (1% glucose, 0.3% yeast extract, and 0.5% peptone). After incubation at 28°C and 180 rpm for 12–16 h, vegetative growth (10% v/v) was transferred to 35 L seed fermentor containing 25 L GYE (1% glucose and 0.3% yeast extract) medium. Ten percent (v/v) of the vegetative growth was used to inoculate the 400 L fermentor containing 300 L medium. Aeration was maintained between 0.8 and 1 vvm. Agitation was kept initially at 70 rpm and slowly increased to reach 80 rpm at 45 h and was kept constant till the end of fermentation (48–55 h). Dissolved oxygen and temperature were maintained at 60–80% and 26–28°C, respectively. Fermentor was terminated when activity started declining and mycelial biomass was separated from the broth by centrifugation in bucket centrifuges. The cell-free supernatant was concentrated by membrane filtration with 10 kDa cut-off (PM-10 membrane) and used for stability studies.

### 2.3. Stability Studies

Effect of additives on thermal stability was determined by incubating the enzyme (1000 unit) preparation in the presence of additives at the desired concentrations and temperatures for a stipulated period of time. Sugars, sugar alcohols, metal ions, salts, and polyethylene glycol were used as additives. Aliquots were withdrawn at regular time intervals and cooled on ice for 15 min and residual activity was estimated at optimum condition 50°C, pH 9 [8]. The activity of enzyme without additive was considered as control and taken as 100%.

### 2.4. Enzyme Assay

Protease activity was determined at 50°C and pH 9 with 1% casein as substrate as described earlier [10] with slight modification. Briefly, the reaction mixture contained 1 mL of suitably diluted enzyme and 1 mL of 1% Hammerstein casein in 0.1 M carbonate-bicarbonate buffer pH 9.0. After incubation at 50°C for 10 min, the reaction was terminated by the addition of 3 mL of 5% trichloroacetic acid. The precipitate formed was filtered through Whatman number 1 filter paper after standing at room temperature for 30 min. The absorbance of trichloroacetic acid soluble fraction was measured at 280 nm. Amount of tyrosine produced is calculated from a precalibrated graph of absorbance at 280 nm against tyrosine concentration. One unit of protease activity was defined as the amount of enzyme liberating 1 *μ*g Tyr/mL/min under assay conditions.

### 2.5. Statistical Analysis

The experiments were run in triplicate. Data were presented as mean values with standard deviations. One way analysis of variance (ANOVA) was carried out and mean comparisons were run using Duncan's multiple range tests.

## 3. Results and Discussion

### 3.1. Thermostability of Protease

**Figure 1:**
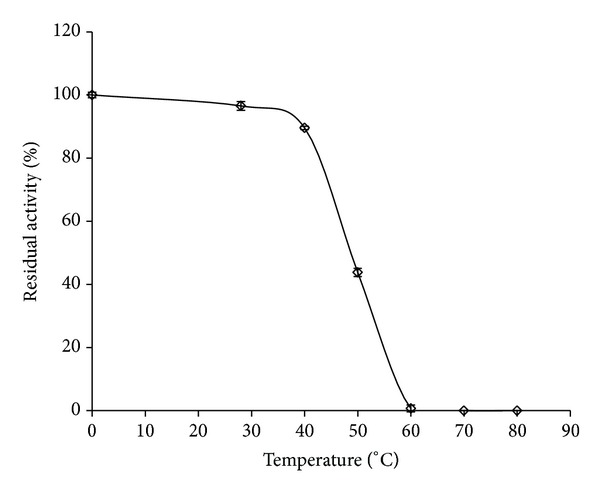
Thermostability of fungal alkaline protease during 1 h incubation at different temperatures. Values are mean ± standard deviation (*n* = 3).

Thermostability of protease was evaluated by incubating the crude enzyme (1000 units) at temperatures ranging from 30 to 80°C for 1 h (Figure 1). The enzyme was stable up to 40°C and around 50% activity was retained at 50°C while complete loss was observed above this temperature. These results are consistent with thermal stabilities of other fungal proteases reported in literature.* Penicillium* protease was not stable above 35°C and residual activities after 1 h at 35, 45, and 50°C were 90, 60, and 20%, respectively [11].* Aspergillus parasiticus* protease was stable up to 40°C for 1 h incubation but was inactivated above this temperature [12]. This is comparable to the present fungal protease which retained around 90% of activity at 40°C after 1 h. The present fungal protease retained more than 65% activity at 28°C during 24 h of incubation (data not shown). These results are consistent with alkaline protease from* Conidiobolus coronatus* which was stable at 28°C for 20 h and at 40°C for 1 h [13].

The alkaline proteinase from* Fusarium culmorum* was heat labile and residual activities after 50 min at 24, 40, 50, and 60°C were 88, 55, 29, and 0%, respectively [14]. Our fungal protease was more thermostable at 50°C (half-life around 50 min) than the proteases from* A*.* clavatus* ES1 (half-life: 30 min) and* A. clavatus* CCT2759 (half-life: 18 min) [15, 16]. The present fungal protease was less stable than* Aspergillus fumigatus* TKU003 protease which retained its initial activity from 25 to 50°C and 47% of its activity at 60°C but was completely inactivated at 70°C after 30 min [17]. However, FAP was optimally active at 50°C [8]. Hence, all further stability studies were carried out at 50°C.

### 3.2. Effect of CaCl_2_ and MgCl_2_

Ca^2+^ is a known activator of protease activity and also offers protection against thermal inactivation. Effect of Ca^2+^ and Mg^2+^ on stability of fungal alkaline protease was studied by incubating the enzyme with varying concentrations of CaCl_2_ and MgCl_2_ (5 to 20 mM) at 50°C up to 3 h (Table 1). In general, residual activity of FAP decreased as the incubation time increased (*P* < 0.05). However, decrease in residual activity was lowered in FAP with additives as compared to control (*P* < 0.05). Ca^2+^ was found to be a better protectant compared to Mg^2+^ at all the concentrations tested. Increasing the metal ion concentration increased the thermostability of FAP at 50°C. 20 mM Ca^2+^ increased the stability by 2-3 times compared to control especially after 60 min. Residual activities at the end of 3 h of incubation at 50°C in control, in presence of 20 mM Ca^2+^ and 20 mM Mg^2+^, were 17.4%, 56.9%, and 41.7%, respectively. The protective effect of 5 mM Ca^2+^  was similar to that with 10 mM Mg^2+^, but effect of 20 mM Mg^2+^ was less than 10 mM Ca^2+^. Similar pattern of increase in thermostability by Ca^2+^ was also observed in alkaline proteases from* P. lilacinus*,* Aspergillus oryxae*,* A. tamarii,* and* Conidiobolus coronatus* [13, 18–20]. 5 mM CaCl_2_ offered maximum protection against thermal inactivation of* A. tamarii *protease increasing the half-life at 55°C from 20 to 140 min. But the protective effect gradually decreased with increasing CaCl_2_ concentration, while the addition of other salts such as MgCl_2_, MnCl_2_, and NaCl did not offer any protection [20]. Addition of 1 mM Ca^2+^ increased the stability of* P. lilacinus *proteinase at 60°C by 2.5 times [18]. Ca^2+^ ion played a vital role in maintaining the active confirmation of the enzyme at higher temperatures [21]. The protective effect of Ca^2+^ against thermal inactivation is speculated to be due to the strengthening of intramolecular interactions in the protein molecule as well as the binding of Ca^2+^ to autolysis sites.

### 3.3. Effect of Glycine

Effect of 0.5 and 1 M glycine was investigated on thermostability of FAP. Glycine at both concentrations (0.5 and 1 M) increased the stability of the enzyme by almost 1.7–2.2 times over control, especially after 1 h of incubation (Table 2). There was no additional benefit in increasing the concentration of glycine beyond 0.5 M as the residual activities for 0.5 and 1 M glycine after 3 h were 31.6 and 35.2, respectively, as compared to 16.11% in control. Addition of 1 M glycine was found to be effective in improving the stability of* C. coronatus* protease which retained 31% of activity after incubation at 50°C for 1 h as compared to control and the half-life of the enzyme increased from 17 to 42 min [13]. Anjum et al. [22] studied the compatibility of osmolytes (glycine, proline, and sarcosine) with the Gibbs energy of stabilization of proteins (on heat induced denaturation of lysozyme, ribonuclease A, cytochrome c, and myoglobin). They observed an increase in *T*_*m*_ (midpoint of the denaturation) with an increase in osmolyte concentration without change in Gibbs energy of stabilization. The main factor responsible for the stabilizing effect was correlated with the preferential exclusion of the osmolytes from the protein domain leading to the protein with lower exposed surface, thereby displacing the denaturation equilibrium towards native state. Thus, osmolytes are known to be effective in increasing thermostability and function by promoting the refolding and reactivation of the thermally unfolded proteins.

### 3.4. Effect of Trehalose

**Figure 2:**
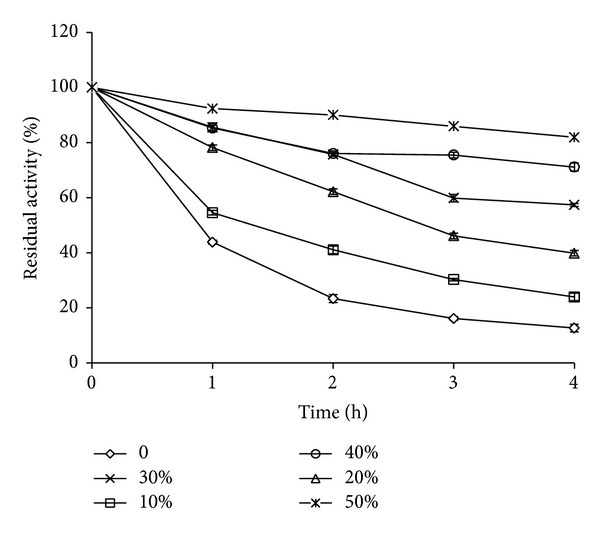
Effect of trehalose on thermostability of fungal alkaline protease during 4 h incubation at 50°C. Values are mean ± standard deviation (*n* = 3).

Effect of trehalose (*α*-D-glucopyranosyl-*α*-D-glucopyranoside), a nonreducing sugar on thermostability of FAP, was investigated in the concentration range of 10 to 50%. Extent of stability of the protease was dependent on trehalose concentration and nearly 2–6-fold increase was observed in comparison with control (Figure 2). More than 80% activity was retained even after 4 h at 50°C in presence of 50% trehalose compared to 12% in controls under identical conditions. At the concentration of 30% trehalose, more than 50% of FAP activity was observed. Trehalose is reported to effectively prevent protein denaturation and aggregation of denatured proteins at high temperatures. It is known to increase the thermostability of an enzyme by changing its microenvironment [23].

Therefore, trehalose plays an important role in preventing protein denaturation at high temperature by changing its microenvironment and suppressing the aggregation of denatured proteins.

### 3.5. Effect of Polyols

**Figure 3:**
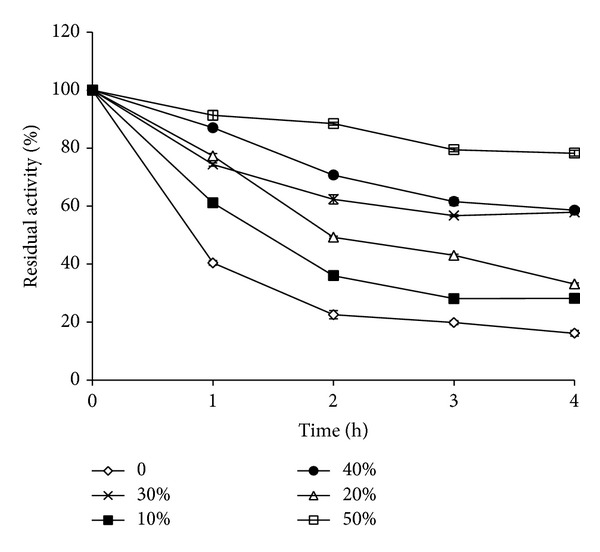
Effect of sorbitol on thermostability of fungal alkaline protease during 4 h incubation at 50°C. Values are mean ± standard deviation (*n* = 3).

Stability of FAP in presence of sugar alcohols including glycerol, mannitol, sorbitol, and xylitol was investigated. All sugar alcohols increased the stability by several folds over control, with the effect being more pronounced where enzyme was incubated for longer times (Table 3). At 10% concentration, the half-life of the enzyme increased from 50 min in control in the absence of additive to 80, 60, 90, and 75 min for glycerol, mannitol, sorbitol, and xylitol, respectively. At 20% concentration, the half-life of the enzyme further increased from 50 min in control to 90, 115, 135, and 95 min for glycerol, mannitol, sorbitol, and xylitol, respectively. Since sugar alcohols were found to be very effective, all the sugar alcohols were also evaluated at 50% which increased the half-lives from 50 min in control to more than 240 min, the longest duration tested (data not shown). It is worth mentioning that 50% sorbitol increased the stability by 4-5 times as compared to control when incubated beyond 1 h with 80% original activity being retained even after 4 h while it was 16% in control under identical conditions (Figure 3). The residual activities after 1 h at 60°C in absence and presence of 50% sorbitol were <1% and 52.9%, respectively. Though 50% sorbitol increased the stability of protease at 60°C, it could not offer protection above this temperature and complete loss in activity was noticed within 1 h even in presence of 50% sorbitol (data not shown). Water plays an important role in influencing the thermostability of enzymes. A range of low-molecular weight additives, such as sugars and polyols, exert stabilizing effect by inducing preferential hydration of proteins. At least in case of polyols, the preferential hydration arises from an increase in the surface tension of solvent water [24]. Loss of the protein's compact, properly folded structure increases the protein solvent interface which in turn tends to increase the degree of thermodynamically unfavourable interaction between the additive and the protein molecule resulting in the stabilization of protein by the additive [22]. 1% glycerol and 800 mM sorbitol offered limited protection against thermal inactivation of* C. coronatus* protease at 50°C [13].

Addition ofglycerol, sucrose, mannitol, sorbitol, and starchincreased the half-life of an alkaline protease from* B mojavensis* at 60°C by 2–2.2-fold [25]. Addition of 3 M sorbitol also improved the thermal stability of* B. cereus* BG1 alkaline protease at 60°C by 2-fold [26]. The protective effects were explained by the strengthening of the hydrophobic interactions inside protein molecules and by indirect action of polyols on water structure.

### 3.6. Effect of PEG 6000

PEG 6000 in concentration range of 5–15% had no beneficial effect on stability of protease. In fact, there was slight decrease in the residual activity at all the concentrations and at all the incubation times tested (Table 4). 10% PEG 8000 offered limited protection against thermal inactivation of* C. coronatus* protease at 50°C [13]. Actual mechanism of stabilization by polyethylene glycols (PEGs) is not yet well understood. It has been suggested that ethylene glycol is likely to stabilize polar proteins, while destabilizing nonpolar ones [23]. The effectiveness of PEG on proteins highly depends on the polymer molecular weight and on the protein structure. PEGs with high molecular weights are believed to prevent protein-protein interactions, leading to stabilization. In the present investigation, PEG 6000 showed slight inactivation/denaturation, and inactivation increased with an increase in PEG added. It suggested that increased hydrocarbon chain affects the protease structure which interferes with its refolding.

### 3.7. Effect of NaCl and K_2_HPO_4_

Inorganic salts have been investigated for their influence on the stability of enzymes [13, 20]. Effect of NaCl (0.5 and 1 M), K_2_HPO_4_ (0.5 and 1 M), and (NH_4_)_2_SO_4_ (5, 10, and 15%) on thermostability of protease at 50°C was investigated. Both 0.5 and 1 M NaCl increased stability of protease by 2 times. Residual activity after 3 h in case of control was around 17. 4%, which increased to 37.4% in presence of NaCl (Table 5). There was no added advantage of 1 M NaCl as residual activities were more or less similar to 0.5 M NaCl. Effect of 0.5 M K_2_HPO_4_ was not very significant but stabilizing effect of 1 M K_2_HPO_4_ was similar to that of 0.5 M NaCl. 0.2 M NaCl had no beneficial effect on the stability of* C. coronatus* protease [13].

**Figure 4:**
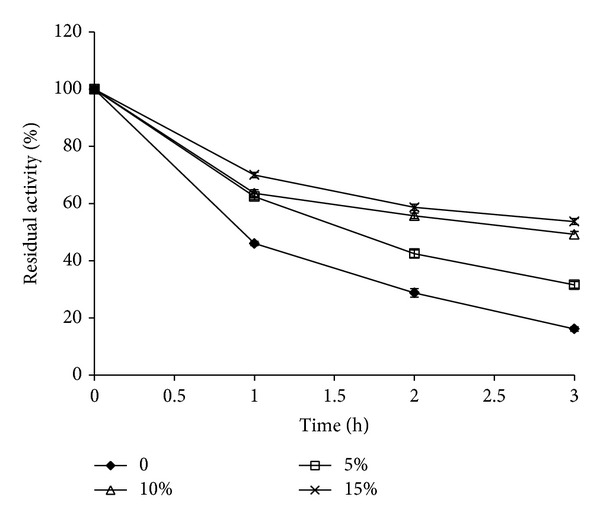
Effect of ammonium sulphate on thermostability of fungal alkaline protease during 3 h incubation at 50°C. Values are mean ± standard deviation (*n* = 3).

Ammonium sulphate, a known stabilizer, was examined for its stabilizing effect in the concentration range of 5 to 15% (concentration at which no precipitation was observed). Ammonium sulphate increased the stability which was found to be concentration dependent (Figure 4). 15% ammonium sulphate increased the stability at 50°C by 3-fold. Salts such as MgCl_2_, MnCl_2_, and NaCl were found to be ineffective in protecting* A. tamarii* protease against thermal inactivation [20]. Salts play an important role in thermostabilization by known mechanism of “salting in” at low concentration; here, it also obeys preferential principle of hydration and addition of 15% ammonium sulphate resulted in 3-fold increase in stability as compared to control.

## 4. Conclusions

The fungal alkaline protease retained more than 60% activity after 24 h at 28°C and 1 h at 40°C and around 50% activity was retained at 50°C, while a complete loss was observed above this temperature. Ca and Mg, trehalose, sugar alcohols, and salts like NaCl and ammonium sulphate offered protection to varying degrees against thermal inactivation at 50°C, while Ca was better than Mg. Among the additives tested, trehalose and the sugar alcohols especially sorbitol were found to be the best stabilizers with 80% residual activity after 4 h incubation at 50°C. Thermal stability increases the efficiency of enzymes and is one of the essential features for their commercial exploitation. The present fungal protease is more thermostable than proteases from* Penicillium* sp.,* Fusarium culmorum*,* A*.* clavatus*,* Beauveria bassiana*,* S. brevicaulis,* and* C. coronatus* but less stable than protease from* Aspergillus tamarii*.

## Figures and Tables

**Table 1:** Effect of CaCl_2_ and MgCl_2_ on thermostability of fungal alkaline protease during 3 h of incubation at 50°C.

Time (min)	Residual activity (%)						
	Control	CaCl_2_ (mM)			MgCl_2_ (mM)		
		5	10	20	5	10	20
0	100	100	100	100	100	100	100
30	73.4 ± 0.91^d^	81.5 ± 1.92^c^	91.7 ± 1.3^b^	96.9 ± 1.64^a^	72.7 ± 0.21^d^	91.9 ± 0.89^b^	91.8 ± 0.64^b^
60	44.4 ± 0.48^f^	61.1 ± 0.65^d^	73.2 ± 0.35^b^	79.8 ± 1.34^a^	54.7 ± 0.72^e^	64.3 ± 1.16^d^	70.3 ± 1.37^c^
120	22.7 ± 0.19^g^	44.0 ± 0.44^e^	63.8 ± 1.00^b^	68.1 ± 1.13^a^	39.0 ± 0.49^f^	47.2 ± 0.48^d^	58.8 ± 0.31^c^
180	17.4 ± 0.38^g^	34.8 ± 0.42^d^	54.6 ± 2.44^b^	56.9 ± 0.89^a^	28.3 ± 0.40^f^	32.2 ± 0.46^e^	41.7 ± 0.41^c^

Values represent mean ± SD from triplicates. Different small letters in the same row indicate significant difference (*P* < 0.05).

**Table 2:** Effect of glycine on thermostability of fungal alkaline protease during 3 h of incubation at 50°C.

Time (min)	Residual activity (%)		
	Control	Glycine (M)	
		0.5	1
0	100	100	100
60	43.8 ± 0.72^c^	54.2 ± 0.60^b^	56.3 ± 0.41^a^
120	23.3 ± 0.38^c^	39.9 ± 0.53^a^	40.1 ± 0.63^a^
180	16.1 ± .043^c^	31.6 ± 0.43^b^	35.2 ± 0.12^a^

Values represent mean ± SD from triplicates. Different small letters in the same row indicate significant difference (*P* < 0.05).

**Table 3:** Effect of different sugar alcohols on thermostability of fungal alkaline protease during 3 h of incubation at 50°C.

Time (Min)	Residual activity (%)								
	Control	Glycerol		Mannitol		Sorbitol		Xylitol	
		10%	20%	10%	20%	10%	20%	10%	20%
0	100	100	100	100	100	100	100	100	100
30	69.7 ± 1.42^d^	68.0 ± 0.31^d^	74.7 ± 1.10^c^	68.1 ± 0.74^d^	74.1 ± 1.72^c^	79.2 ± 1.78^b^	84.0 ± 1.84^a^	68.1 ± 2.08^d^	74.3 ± 1.72^c^
60	43.6 ± 0.93^e^	55.9 ± 1.26^d^	62.0 ± 1.66^b^	54.1 ± 0.88^d^	64.4 ± 0.15^b^	59.7 ± 0.09^c^	70.7 ± 0.58^a^	55.3 ± 0.17^d^	63.4 ± 0.31^b^
120	23.8 ± 0.25^f^	35.3 ± 0.65^e^	37.0 ± 0.46^d^	33.6 ± 0.61^e^	48.7 ± 0.31^b^	42.5 ± 0.50^c^	51.6 ± 0.39^a^	38.5 ± 0.68^d^	44.6 ± 1.07^c^
180	17.0 ± 0.10^e^	28.0 ± 0.19^d^	31.7 ± 0.26^c^	27.2 ± 0.47^d^	40.7 ± 0.46^b^	31.1 ± 0.21^c^	47.1 ± 0.55^a^	29.3 ± 0.19^d^	39.2 ± 0.48^b^

Values represent mean ± SD from triplicates. Different small letters in the same row indicate significant difference (*P* < 0.05).

**Table 4:** Effect of PEG 6000 onthermostability of fungal alkaline protease during 3 h of incubation at 50°C.

Time (min)	Residual activity (%)			
	Control	PEG (%)		
		5	10	15
0	100	100	100	100
30	68.0 ± 1.17^a^	52.4 ± 0.75^b^	53.6 ± 2.15^b^	38.9 ± 1.02^c^
60	43.2 ± 0.20^a^	37.9 ± 1.03^b^	32.9 ± 0.29^c^	19.1 ± 0.16^d^
120	22.5 ± 0.05^a^	21.2 ± 0.50^a^	18.6 ± 0.29^b^	11.6 ± 0.18^c^
180	16.7 ± 0.12^a^	12.4 ± 0.05^b^	10.9 ± 0.30^c^	8.5 ± 0.14^d^

Values represent mean ± SD from triplicates. Different small letters in the same row indicate significant difference (*P* < 0.05).

**Table 5:** Effect of NaCl and K_2_HPO_4_ on thermostability of fungal alkaline protease during 3 h of incubation at 50°C.

Time (min)	Residual activity (%)				
	Control	NaCl (M)		K_2_HPO_4_ (M)	
		0.5	1	0.5	1
0	100	100	100	100	100
30	70.4 ± 0.91^c^	77.8 ± 0.71^b^	80.5 ± 0.57^a^	70.1 ± 1.85^c^	71.3 ± 0.80^c^
60	44.4 ± 0.48^d^	54.1 ± 1.16^b^	62.0 ± 0.53^a^	50.0 ± 0.71^c^	57.5 ± 0.94^b^
120	21.8 ± 0.58^d^	48.8 ± 0.57^a^	49.2 ± 0.25^a^	36.7 ± 0.13^c^	47.1 ± 0.31^b^
180	17.4 ± 0.38^d^	37.4 ± 0.30^a^	37.6 ± 0.62^a^	24.9 ± 0.22^c^	28.7 ± 0.14^b^

Values represent mean ± SD from triplicates. Different small letters in the same row indicate significant difference (*P* < 0.05).
